# Concomitant Release of Ventral Tegmental Acetylcholine and Accumbal Dopamine by Ghrelin in Rats

**DOI:** 10.1371/journal.pone.0049557

**Published:** 2012-11-14

**Authors:** Elisabet Jerlhag, Anna Carin Janson, Susanna Waters, Jörgen A. Engel

**Affiliations:** 1 Section for Pharmacology, Institute of Neuroscience and Physiology, The Sahlgrenska Academy at the University of Gothenburg, Gothenburg, Sweden; 2 NeuroSearch Sweden AB, Gothenburg, Sweden; Hokkaido University, Japan

## Abstract

Ghrelin, an orexigenic peptide, regulates energy balance specifically via hypothalamic circuits. Growing evidence suggest that ghrelin increases the incentive value of motivated behaviours via activation of the cholinergic-dopaminergic reward link. It encompasses the cholinergic afferent projection from the laterodorsal tegmental area (LDTg) to the dopaminergic cells of the ventral tegmental area (VTA) and the mesolimbic dopamine system projecting from the VTA to nucleus accumbens (N.Acc.). Ghrelin receptors (GHS-R1A) are expressed in these reward nodes and ghrelin administration into the LDTg increases accumbal dopamine, an effect involving nicotinic acetylcholine receptors in the VTA. The present series of experiments were undertaken directly to test this hypothesis. Here we show that ghrelin, administered peripherally or locally into the LDTg concomitantly increases ventral tegmental acetylcholine as well as accumbal dopamine release. A GHS-R1A antagonist blocks this synchronous neurotransmitter release induced by peripheral ghrelin. In addition, local perfusion of the unselective nicotinic antagonist mecamylamine into the VTA blocks the ability of ghrelin (administered into the LDTg) to increase N.Acc.-dopamine, but not VTA-acetylcholine. Collectively our data indicate that ghrelin activates the LDTg causing a release of acetylcholine in the VTA, which in turn activates local nicotinic acetylcholine receptors causing a release of accumbal dopamine. Given that a dysfunction in the cholinergic-dopaminergic reward system is involved in addictive behaviours, including compulsive overeating and alcohol use disorder, and that hyperghrelinemia is associated with such addictive behaviours, ghrelin-responsive circuits may serve as a novel pharmacological target for treatment of alcohol use disorder as well as binge eating.

## Introduction

The cholinergic-dopaminergic reward link is an important part of the reward systems [1], [2]. This link encompasses the cholinergic afferent projection from the laterodorsal tegmental area (LDTg) to the dopaminergic cells in the ventral tegmental area (VTA) and the mesolimbic dopamine system projecting from the VTA to the nucleus accumbens (N.Acc.) [3], [4], [5]. In addition to increasing accumbal dopamine release, natural rewards and dependence-producing drugs simultaneously enhance VTA acetylcholine levels [6], [7], [8], [9], implicating that this reward link may have a role in the hedonic aspects of rewards, natural as well as artificial. A disruption in the reward systems underlies, at least in part, addictive behaviours such as alcohol use disorders and binge eating [10], [11], [12]. Common neurobiological mechanisms may be involved in development of both disorders [13].

Growing evidence suggest that ghrelin, a 28 amino acid gut-brain signal, which regulates energy balance specifically via hypothalamic circuits [14], [15], [16], [17], [18], activates this cholinergic-dopaminergic reward link. Indeed, ghrelin receptors (GHS-R1A) are expressed in the reward nodes VTA as well as LDTg [19], [20], [21] and ghrelin administration into these reward nodes increases accumbal dopamine and increases locomotor activity in mice [22], [23], [24]. Ghrelin may, via activation of this reward link, increase the incentive value of motivated behaviours such as reward seeking [25]. The ability of ghrelin to activate the mesolimbic dopamine system, as measured by locomotor activity and accumbal dopamine release, is mediated via nicotinic acetylcholine receptors in the VTA, in mice [1], [24]. Collectively this raises the possibility that ghrelin increases the extracellular levels of ventral tegmental acetylcholine which via activation of nicotinic acetylcholine receptors causes a release of accumbal dopamine. The present series of experiments were undertaken to directly test this hypothesis by investigating the effect of ghrelin (systemically or locally into the LDTg) on VTA-acetylcholine and N.Acc.-dopamine release in one and the same awake, freely moving rat by using *in vivo* microdialysis. In addition, the role of nicotinic acetylcholine receptors, in the VTA, for this activation was investigated.

## Materials and Methods

### Animals

Adult post-pubertal age-matched male Wistar rats (250–300 g body weight; Charles River, Sulzfeld, Germany) were used as a similar study has been reported using this strain [7]. All rats were maintained at 20°C with 50% humidity and a 12/12 hour light/dark cycle (lights on at seven am) and were allowed to habituate at the animal facilities at the EBM (Gothenburg, Sweden) for at least one week before initiation of the experiment. Tap water and food (Normal chow; Harlan Teklad, Norfolk, England) were supplied *ad libitum,* except during the microdialysis experiment. The Ethics Committee for Animal Experiments in Gothenburg, Sweden, has approved the experiments (permit number: 81–07 and 26–12) and all efforts were made to minimize suffering.

### Drugs

Acylated rat ghrelin (Bionuclear; Bromma, Sweden) was diluted in 0.9% sodium chloride (saline vehicle) for peripheral (intraperitoneally, i.p.) (5 ml/kg body weight) administration. The higher dose, 0.33 mg/kg, was selected since it previously has been shown to increase locomotor activity and accumbal dopamine release, induce a conditioned place preference in mice [26] and to increase the consumption [27] as well as the motivation to consume [28] sucrose in rats. The lower dose (0.167 mg/kg) was selected since it previously has been shown to release dopamine in the shell of N.Acc. in rats [29]. For local administrations into the LDTg, ghrelin was dissolved in vehicle solution (Ringer) (NaCl 140 mM, CaCl2 1.2 mM, KCl 3.0 mM and MgCl2 1.0 mM; Merck KGaA, Darmstadt, Germany) and was administered at a dose of 1 µg in 0.5 µl since this dose has been shown to activate the reward systems in mice previously [22]. The selected dose of JMV2959, a GHS-R1A antagonist provided by Æterna Zentaris GbmH, Frankfurt am Main, Germany, was also determined previously (3 mg/kg, i.p.) [27], [30]. JMV2959 was always administered twenty minutes prior to drug (ghrelin or vehicle) exposure. It was diluted in 0.9% sodium chloride (saline vehicle). Indeed, it has been established that JMV2959, when administered peripherally, is a GHS-R1A antagonist and suppresses food intake induced by ghrelin or by the GHS-R1A agonist, hexarelin [31], [32]. Radioligand binding studies have established that JMV2959 is a GHS-R1A antagonist [31] and that it does not affect the dopamine receptors (D1, D2L and D2S receptors) [33]. Mecamylamine hydrochloride (Sigma-Aldrich Sweden AB, Stockholm, Sweden), an unselective nicotinic acetylcholine receptor antagonist, was dissolved in vehicle solution (Ringer) and was perfused locally into the VTA via the microdialysis probe (300 µM during 40 minutes). Ghrelin was administered locally into the LDTg 10 minutes after mecamylamine perfusion was finished. The dose used for mecamylamine was based on dos-response experiments (data not shown). All drug challenges were part of a balanced design with regard to both the treatment order and the number of subjects per treatment. Each rat was only included in one microdialysis experiment.

### *In vivo* Microdialysis

For measurements of extracellular levels (reflecting the release of the neurotransmitter) of dopamine in the N.Acc. and acetylcholine in the VTA, rats were implanted unilaterally with microdialysis probes positioned in the N.Acc. as well as in the VTA. Only rats with correct probe positions in the VTA as well as N.Acc. were included in the statistical analysis. The surgery was preformed as described thoroughly elsewhere [7]. In brief, the rats were anesthetized with isofluran (Isofluran Baxter; Univentor 400 Anaesthesia Unit, Univentor Ldt., Zejtun, Malta), placed in a stereotaxic frame (David Kopf Instruments; Tujunga, CA, USA) and kept on a heating pad to prevent hypothermia. The scull bone was exposed and two holes for the probes and one for the anchoring screw were drilled. The probes were randomly alternated to either the left or right side and were always positioned ipsilateral. The following coordinates were used for N.Acc.: 1.85 mm anterior to the bregma, ±1.0 mm lateral to the midline and 7.8 mm below the surface of the brain surface, and for VTA: 6.0 mm posterior to the bregma, ±0.6 mm lateral to the midline and 8.5 mm below the surface of the brain surface [34]. The probes were attached to the scull with dental cement (Agntho’s AB, Lidingö. Sweden). The exposed tip of the dialysis membrane (20 000 kDa cut off with an o.d./i.d. of 310/220 µm, HOSPAL, Gambro, Lund, Sweden) of the probe was 2 mm for N.Acc. and 1.5 mm for VTA. All probes were surgically implanted two days prior to the experiment. After surgery the rats were kept in individual cages (Macrolon III). To enable local administrations of ghrelin into the LDTg rats were implanted with one unilateral guide cannula aiming at the LDTg. The coordinates for the LDTg were: 8.8 mm posterior to the bregma, ±1.0 mm lateral to the midline and 1 mm below the surface of the brain surface. At the time of the experiment a cannula for drug administration was inserted and extended another 6.0 mm ventrally beyond the tip of the guide cannula [34].

The microdialysis technique enables measurements of neurotransmitters in awake, freely moving animals. On the day of the experiment the probe was connected to a microperfusion pump (U-864 Syringe Pump; AgnThós AB) and perfused with Ringer solution at a rate of 1.6 µl/minute. After one hour of habituation to the microdialysis set-up, perfusion samples were collected every 20 minutes. Neostigmine bromid (Sigma Sigma-Aldrich, Stockholm, Sweden), an acetylcholine esterase inhibitor, was added to the Ringer perfusion in the VTA throughout the entire experiment at a concentration of 0.5 µM to improve the detection of acetylcholine. It has previously been shown that this dose does not affect the extracellular concentrations of dopamine in the N.Acc. [7]. However, it cannot be excluded that this manipulation may interfere with the experimental conditions.

The first series of experiments were undertaken to investigate if peripheral ghrelin activates the cholinergic-dopaminergic reward link in the brain. The effects of two different doses of ghrelin (0.167 or 0.33 mg/kg i.p.) compared to vehicle (sodium chloride) on ventral tegmental acetylcholine and accumbal dopamine in rats were investigated. The doses used are from a narrow dose interval and the experiment should therefore not be considered as a dose response study. The lower dose (0.167 mg/kg) has previously been shown to release dopamine in the shell of N.Acc. in rats [29] and the higher dose (0.33 mg/kg) has been found to activate the mesolimbic dopamine system in mice [26] and to increase the consumption and motivation to consume sucrose in rats [27], [28]. The baseline acetylcholine or dopamine level was defined as the average of three consecutive samples before the drug challenge, and the increase in acetylcholine or dopamine was calculated as the percentage increase from baseline. After the baseline samples, rats were injected with either a high dose of ghrelin (0.33 mg/kg), low dose of ghrelin (0.167 mg/kg) or vehicle. The following eight 20-minute samples were collected and analysed.

The second series of experiments were conducted to confirm that the ability of ghrelin to activate the cholinergic-dopaminergic reward link is mediated via GHS-R1A. The higher dose of ghrelin (0.33 mg/kg i.p.) was used since this dose is commonly used in rats and produces a robust activation of the cholinergic-dopaminergic reward link. In these experiments, conducted in separate rats, we examine the effect that the GHS-R1A antagonist JMV2959 has on ghrelin-induced release of acetylcholine and dopamine in the VTA and N.Acc., respectively. The baseline acetylcholine or dopamine levels were defined as the average of three consecutive samples before the first drug challenge, and the increase in acetylcholine or dopamine was calculated as the percentage increase from baseline. After the baseline samples, rats were injected with JMV2959 (3 mg/kg, i.p.) or vehicle. 20 minutes later this was followed by a ghrelin (0.33 mg/kg, i.p.) or vehicle injection, creating four treatment groups, *i.e.* vehicle-vehicle; vehicle-ghrelin; JMV2959-vehicle; JMV2959-ghrelin. The following eight samples were collected and analysed.

In subsequent experiments we investigated if ghrelin locally into the LDTg activates the cholinergic-dopaminergic reward link and if this activation is mediated via local nicotinic acetylcholine receptors in the VTA. Therefore, we examined whether ghrelin administered locally into the LDTg produces a concomitant release of acetylcholine in the VTA and dopamine in the N.Acc. in rats. In addition, we investigated the effect of local perfusion of the unselective nicotinic acetylcholine receptor antagonist, mecamylamine, into the VTA on ghrelin (administered into the LDTg) induced synchronous neurotransmitter release. The baseline acetylcholine or dopamine levels were defined as the average of three consecutive samples before the first drug challenge. Mecamylamine (300 µM) or vehicle (Ringer) was perfused locally into the VTA via the probe for 40 minutes and thereafter ghrelin (1 µg in 0.5 µl) or vehicle (Ringer) was administered locally into the LDTg, creating four treatment groups, *i.e.* vehicle-vehicle; vehicle-ghrelin; mecamylamine-vehicle; mecamylamine-ghrelin. The increase in acetylcholine or dopamine was calculated as the percentage increase from baseline. The following eight samples were collected and analysed.

### Verification of Probe and Cannula Placement

After the microdialysis experiments were completed, the locations of the probes and guide cannula were verified. The rats were decapitated, probes were perfused with pontamine sky blue 6BX to facilitate probe localization, and the brains were mounted on a vibroslice device (752M Vibroslice; Campden Instruments Ltd., Loughborough, UK). The brains were cut in 50 µm sections and the location of the probes and guide cannula were determined by gross observation using light microscopy. The exact positions were verified [34]. It should be emphasized that only rats with correct probe positions in the VTA, i.e. for acetylcholine measurements, as well as N.Acc., i.e. for dopamine measurements, and rats with correct guide cannula positions in the LDTg (for the third series of experiments) were included in the statistical analysis. In all experiments a total of 14 rats had the probe misplaced in either the VTA or N.Acc. and these rats were not included in the statistical analysis.

### Acetylcholine Analysis

The acetylcholine levels in the dialysates were determined by means of liquid chromatography/tandem mass spectrometry (LC-MS/MS), essentially as described elsewhere [35], [36]. In brief, liquid chromatography was performed using a Hewlett-Packard 1100 Series system. The module includes a binary pump system, a vacuum degasser, a thermostated autosampler and a thermostated column (Kinetex 2.6 u HILIC 100A 100*2.1 mm). Flow: 0.25 ml/min, column temperature: 20°C, injection volume: 5 µl. The mobile phase was A: 10/10/80, 200 mM ammonium formate pH:3.5/acetonitrile/water and B: 10/80/10, 200 mM ammonium formate pH:3.5/acetonitrile/water, starting with 40 to 0% A for 2 minutes, followed by 0% A for 4 minutes and then 0–40% A for 2 minutes. Analysis was performed by multiple reaction monitoring (MRM), using a quadrupole linear-ion-trap mass spectrometer (Sciex Q-Trap) equipped with a turboionspray source with ionization in positive mode. The following transitions were monitored: Acetylcholine m/z 146 → m/z 87 and Acetyl-ß-methylcholine (IS) m/z 160 → m/z 101. For calibration stock samples was used. Stock solutions of acetylcholine and acetyl-ß-methylcholine (IS) were prepared at concentrations of 1 mM by dissolving them in Milli-Q water. These solutions were further diluted with water to obtain working solutions (stored at 4°C). IS was prepared in a concentration of 200 nM in Milli-Q water. A calibration curve over the concentration range of 0.5–100 nM for acetylcholine was prepared by adding appropriate amounts of acetylcholine to Ringer solution. Analysis calibration samples (25 µl) were prepared in autosampler vials by adding 5 µl of IS. Data handling was performed with the Analyst 1.4.1. software.

### Dopamine Analysis

The dopamine levels in the dialysates were determined by means of HPLC with electrochemical detection (HPLC-EC). The analysis was preformed as described thoroughly elsewhere [1], [7]. In brief, a pump (Gyncotec P580A; Kovalent AB), an ion exchange column (2.0×100 mm, Prodigy 3 µm SA; Skandinaviska GeneTec AB; Kungsbacka, Sweden) and a detector (Antec Decade; Antec Leyden, Zoeterwoude, The Netherlands) equipped with a VT-03 flow cell (Antec Leyden) were used. The mobile phase (pH 5.6), consisting of sulfonic acid 10 mM, citric acid 200 mM, sodium citrate 200 mM, 10% EDTA, 30% methanol, was vacuum filtered by using a 0.2 µm membrane filter (GH Polypro; PALL Gelman Laboratory, Lund, Sweden). The mobile phase was delivered at a flow rate of 0.2 ml/min passing a degasser (Kovalent AB), and the analyte was oxidized at +0.4 V.

### Statistical Analyses

All microdialysis experiments were evaluated by a two-way analysis of variance (ANOVA) followed by Bonferroni post-hoc test for comparisons between different treatments and specifically at given time points. Data are presented as mean ± SEM.

## Results

### Effects of Peripheral Ghrelin on Concomitant Ventral Tegmental Acetylcholine and Accumbal Dopamine Release in Rats

Here we initially showed that ghrelin (0.167 and 0.33 mg/kg) increases ventral tegmental and accumbal dopamine concomitantly in rats.

Both doses of ghrelin (0.167 and 0.33 mg/kg) increased ventral tegmental acetylcholine release relative to vehicle treatment (*P* = 0.0105 and P = 0.0015, respectively) (treatment F(2,16) = 8.42, *P* = 0.0032; time F(12,192) = 2.54, *P* = 0.0040; treatment x time interaction F(12,192) = 1.68, *P* = 0.0305). This increase was significant at time interval 20 minutes (*P*<0.01) and 100 minutes (P<0.05) (for ghrelin 0.33 mg/kg versus vehicle). No statistically significant difference was observed between the two different doses of ghrelin (0.33 mg/kg and 0.167 mg/kg) (P = 0.3212) (data not shown).

Both doses of ghrelin (0.167 and 0.33 mg/kg) increased accumbal dopamine release relative to vehicle treatment (*P* = 0.0062 and P = 0.0003 respectively) (treatment F(2,16) = 11.56, *P* = 0.0008; time F(12,192) = 3.26, *P* = 0.0003; treatment x time interaction F(12,192) = 2.64, *P* = 0.0001). This increase was significant at time intervals 60–80 minutes (*P*<0.001), 140 minutes (P<0.01) and 180 minutes (*P*<0.05) (for ghrelin 0.33 mg/kg versus vehicle). No statistically significant difference was observed between the two different doses of ghrelin (0.33 mg/kg and 0.167 mg/kg) (P = 0.1473) (data not shown).

Given that acetylcholine and dopamine was measured in one and the same rats the number of rats induced in the statistical analysis was identical (n = 8 for vehicle, n = 6 for ghrelin 0.167 mg/kg, n = 5 for ghrelin 0.33 mg/kg).

### Effects of GHS-R1A Antagonist on Peripheral Ghrelin-induced Ventral Tegmental Acetylcholine and Accumbal Dopamine Release in Rats

**Figure 1:**
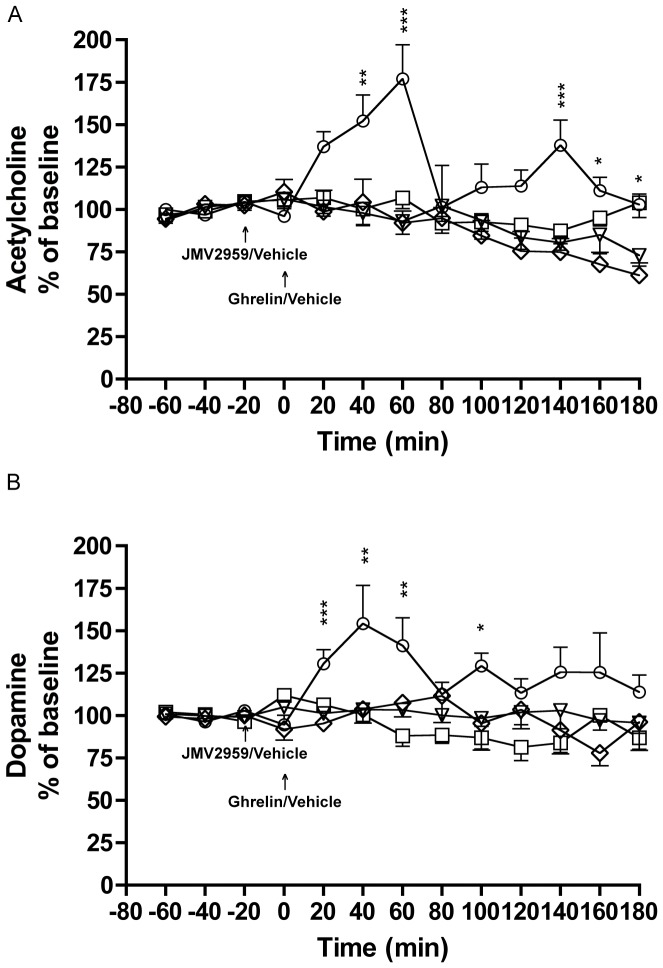
A ghrelin receptor antagonist attenuates ghrelin-induced ventral tegmental acetylcholine and accumbal dopamine release in rats. A) Ghrelin (0.33 mg/kg) increased ventral tegmental acetylcholine release relative to vehicle treatment and this increase was attenuated by pre-treatment with JMV2959 (3 mg/kg, i.p.). No difference was observed between vehicle-vehicle and JMV2959-ghrelin treatment and JMV2959 had no effect on acetylcholine release per se. B) Ghrelin (0.33 mg/kg) increased accumbal dopamine release relative to vehicle treatment and this increase was attenuated by pre-treatment with JMV2959. No difference was observed between vehicle-vehicle and JMV2959-ghrelin treatment and JMV2959 had no effect on dopamine release per se (n = 8 for vehicle-vehicle (square), n = 5 for vehicle-ghrelin (circle), n = 6 for JMV2959-vehicle (rhomb), n = 7 for JMV2959-ghrelin (triangle). All values represent mean ± SEM (***P<0.001, **P<0.01 and *P<0.05).

The second series of experiments first verified our initial findings that ghrelin concomitantly increases ventral tegmental acetylcholine and accumbal dopamine, and secondly showed that a GHS-R1A antagonist blocked this dual neurotransmitter release(Figure 1A and 1B).

Thus, these experiments showed that ghrelin (0.33 mg/kg) increased ventral tegmental acetylcholine release relative to vehicle treatment (*P* = 0.0023) and that this increase was attenuated by pre-treatment with JMV2959 (3 mg/kg) (*P* = 0.0003) (treatment F(3,22) = 8.63, *P* = 0.0006; time F(12,264) = 6.43, *P*<0.0001; treatment x time interaction F(12,264) = 3.30, *P*<0.0001). This attenuation was significant at time intervals 40 minutes (*P*<0.001), 60 and 140 minutes (*P*<0.001), 160–180 minutes (*P*<0.05) (for vehicle-ghrelin versus JMV2959-ghrelin). No difference was observed between vehicle-vehicle and JMV2959-ghrelin treatment (P = 0.3365). JMV2959 had no effect on acetylcholine release *per se* (*P* = 0.1116) (Figure 1A).

Given that acetylcholine and dopamine was measured in one and the same rats the number of rats induced in the statistical analysis was identical (n = 8 for vehicle-vehicle, n = 5 for vehicle-ghrelin, n = 6 for JMV2959-vehicle, n = 7 for JMV2959-ghrelin).

Ghrelin (0.33 mg/kg) increased accumbal dopamine release relative to vehicle treatment (*P*<0.0011) and this increase was attenuated by pre-treatment with JMV2959 (3 mg/kg) (*P* = 0.0120) (treatment F(3,22) = 5.10, *P* = 0.0079; time F(12,264) = 2.25, *P* = 0.0101; treatment x time interaction F(12,264) = 2.57, *P*<0.0001). This attenuation was significant at time interval 20 minutes (*P*<0.001), 40–60 minutes (*P*<0.01), and 100 minutes (*P*<0.05) (for vehicle-ghrelin versus JMV2959-ghrelin). No difference was observed between vehicle-vehicle and JMV2959-ghrelin treatment (P = 0.3205). JMV2959 had no effect on dopamine release *per se* (*P* = 0.6284) (Figure 1A).

### Effects of an Unselective Nicotinic Acetylcholine Receptor Antagonist Perfused into the VTA and Ghrelin into the LDTg on Concomitant Ventral Tegmental Acetylcholine and Accumbal Dopamine Release in Rats

**Figure 2:**
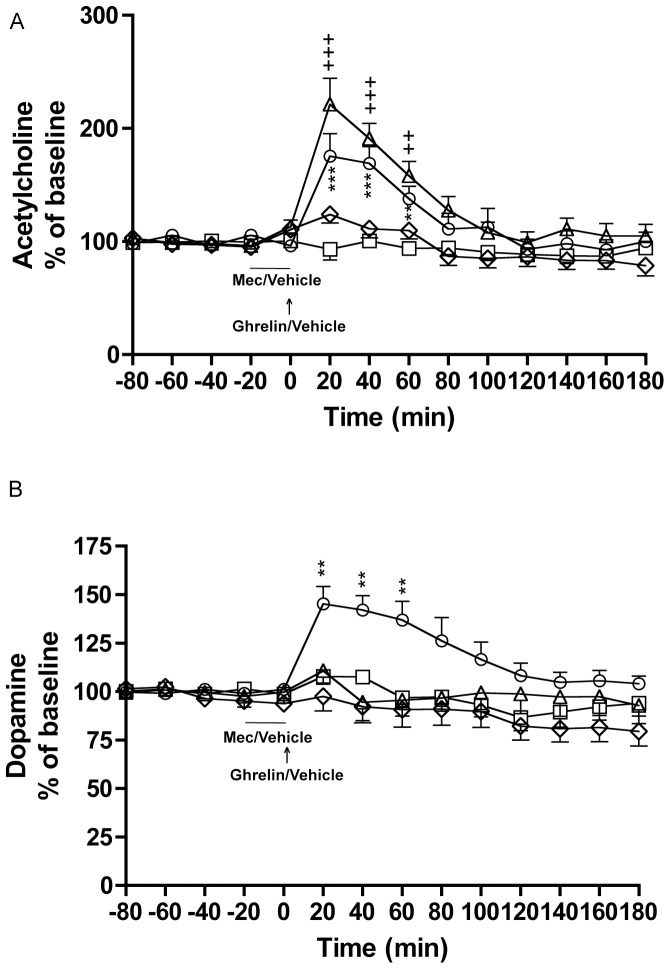
Ghrelin (into the LDTg) increase VTA-acetylcholine and N.Acc.-dopamine and this involves nicotinic acetylcholine receptors in the VTA. A) Ghrelin (locally administered into the LDTg at a dose of 1 µg in 0.5 µl) increased ventral tegmental acetylcholine release relative to vehicle treatment. Pre-treatment with the unselective nicotinic acetylcholine receptor antagonist, mecamylamine, locally into the VTA (300 µM) did not affect the ability of ghrelin to increase VTA-acetylcholine. Mecamylamine had no effect on acetylcholine release per se. B) Ghrelin (locally administered into the LDTg at a dose of 1 µg in 0.5 µl) increased accumbal dopamine release relative to vehicle treatment. This increase was attenuated by pre-treatment with the unselective nicotinic acetylcholine receptor antagonist, mecamylamine, locally into the VTA (300 µM). Mecamylamine had no effect on dopamine release per se (n = 10 for vehicle-vehicle (square), n = 11 for vehicle-ghrelin (circle), n = 11 for mecamylamine-vehicle (rhomb), n = 11 for mecamylamine-ghrelin (triangle). All values represent mean ± SEM (***P<0.001, **P<0.01 for vehicle-vehicle versus vehicle-ghrelin and (+++P<0.001, ++P<0.01 for vehicle-vehicle versus mecamylamine-ghrelin).

Here we initially showed that ghrelin locally into the LDTg increases ventral tegmental and accumbal dopamine concomitantly in rats. In addition we showed that the unselective nicotinic acetylcholine receptor antagonist mecamylamine locally perfused into the VTA blocks the ability of ghrelin (administered into the LDTg) to increase accumbal dopamine, but not ghrelin induced acetylcholine release in the VTA (Figure 2A and 2B).

These experiments showed that local administration of ghrelin into the LDTg (1 µg in 0.5 µl) increased ventral tegmental acetylcholine release relative to vehicle treatment (*P* = 0.0435, vehicle-vehicle vs. vehicle-ghrelin)(treatment F(3,39) = 3.85, *P* = 0.0166; time F(14,546) = 24.29, *P*<0.0001; treatment x time interaction F(14,546) = 4.83, *P*<0.0001). The ability of ghrelin administered into the LDTg to increase ventral tegmental acetylcholine release was significant at time intervals 20–40 minutes (*P*<0.001) and at 60 minutes (*P*<0.01) (vehicle-vehicle versus vehicle-ghrelin). Pre-treatment of mecamylamine into the VTA did not affect the ability of ghrelin into the LDTg to increase ventral tegmental acetylcholine release (*P* = 0.0056, vehicle-vehicle vs. mecamylamine-ghrelin and *P* = 0.3927, vehicle-ghrelin vs. mecamylamine-ghrelin) and this was significant at time intervals 20–40 minutes (*P*<0.001) and at 60 minutes (*P*<0.01) (vehicle-vehicle versus mecamylamine-ghrelin). Mecamylamine had no effect on acetylcholine release *per se* (*P* = 0.6580) (Figure 2A).

These experiments showed that local administration of ghrelin into the LDTg (1 µg in 0.5 µl) increased accumbal dopamine release relative to vehicle treatment (*P* = 0.0207, vehicle-vehicle vs. vehicle-ghrelin)(treatment F(3,39) = 5.11, *P* = 0.0044; time F(14,546) = 6.31, *P*<0.0001; treatment x time interaction F(14,546) = 2.52, *P*<0.0001). This was significant at time interval 20–60 minutes (*P*<0.01) (vehicle-vehicle versus vehicle-ghrelin). This increase was attenuated by pre-treatment with mecamylamine (300 µM) (*P* = 0.0469, vehicle-ghrelin vs. mecamylamine-ghrelin and *P* = 0.6846, vehicle-vehicle vs. mecamylamine-ghrelin). This attenuation was significant at time intervals 20 minute (*P*<0.01), 40 minute (*P*<0.001) and 60 minute intervals (*P*<0.05) (vehicle-ghrelin versus mecamylamine-ghrelin). Mecamylamine had no effect on dopamine release *per se* (*P* = 0.1803) (Figure 2B).

Given that acetylcholine and dopamine was measured in one and the same rats the number of rats induced in the statistical analysis was identical (n = 10 for vehicle-vehicle, n = 11 for vehicle-ghrelin, n = 11 for mecamylamine-vehicle, n = 11 for mecamylamine-ghrelin).

## Discussion

In the present series of experiments we show for the first time that systemic ghrelin administration increases ventral tegmental acetylcholine as well as accumbal dopamine release concomitantly in rats. Peripheral administration of the GHS-R1A antagonist, JMV2959, blocks this synchronous neurotransmitter release, suggesting that ghrelin reaches the brain and activates the cholinergic-dopaminergic reward link via GHS-R1A. In addition, local ghrelin administration into the LDTg increases VTA-acetylcholine and N.Acc.-dopamine; perfusion of the unselective nicotine acetylcholine receptor antagonist (mecamylamine) into the VTA blocks the ghrelin-induced release of accumbal dopamine, but not that of acetylcholine. Collectively, these data indicate that ghrelin activates the LDTg causing a release of acetylcholine in the VTA, which via activation of local nicotinic acetylcholine receptors, causes a release of accumbal dopamine.

Growing evidence has implied that the cholinergic-dopaminergic reward link mediates the reinforcing properties of natural as well as artificial rewards. Specifically, infusion of a nicotinic agonist into the VTA increased the extracellular dopamine levels in the N.Acc. [37] as well as induced conditioned place preference [38]. Food or water intake, not only increases accumbal dopamine release, but also simultaneously enhances the extracellular levels of acetylcholine in the VTA [6], [8], [9]. In addition, lesion of the cholinergic projections from the LDTg to the VTA decreases nicotine and cocaine self-administration [6], [39], inhibits the motivational effects of opiates [40] and attenuates the intake of saccharin and water in rats [41], blocks the rewarding properties of food [40] and impairs sexual activity in naïve male rats [42]. Furthermore, it has been shown that alcohol intake in high alcohol-preferring rats causes a concomitant increase in ventral tegmental acetylcholine and accumbal dopamine [7]. In addition to the LDTg, the caudal compartment of the pedunculopontine tegmental area projects cholinergic afferents to the VTA [43]. Lesion of the caudal part of the pedunculopontine tegmental area decreases nicotine, saccharin and cocaine self-administration [6], [39], [41] and blocks the rewarding properties of food, morphine and amphetamine [40], [44]. Taken together this raises the possibility of a role in reward regulation for other cholinergic afferents to the VTA than those from the LDTg. Here, we provided data showing that ghrelin (administered peripherally or locally into the LDTg) increases ventral tegmental acetylcholine as well as accumbal dopamine, indicating that ghrelin activates the cholinergic-dopaminergic reward link. This hypothesis originated from the data showing that ghrelin administration into the VTA or LDTg increases accumbal dopamine and increases the locomotor activity, effects that are antagonized by nicotinic receptor antagonists administered into the VTA [24]. Ghrelin may, via activation of this reward link, increase the incentive value of motivated behaviours such as reward seeking. Indeed, pharmacological suppression of the GHS-R1A reduces alcohol intake in mice [45], [46] as well as high alcohol consumption and operant lever pressing for alcohol in rats [30]. Furthermore, central ghrelin signalling mediates reward induced by addictive drugs such as alcohol, cocaine, amphetamine as well as nicotine in rodents [33], [45], [47], [48], [49], [50], [51], [52], [53]. GHS-R1A antagonism also attenuates the consumption and motivation to consume palatable foods as well as sweets in rodents [27], [28], [54], [55], [56]. It may be hypothesized that ghrelin signalling could be involved in the process underlying the development of addictive behaviours. Recently, the GHS-R1A were shown to alter the sensitivity of neurons by heterodimerizing with e.g. dopamine D1-like as well as D2 receptors [57], [58]. Taken together with the present and previous studies regarding ghrelin and reward activation it may be postulated that GHS-R1A, via heterodimerization at the level of the cholinergic-dopaminergic reward link, regulate the sensitivity of this reward link and thereby alter the ability of drugs of abuse to activate the reward systems.

The GHS-R1A antagonist, JMV2959, blocks the ghrelin-induced dual increase of acetylcholine in the VTA and dopamine in the N.Acc., implying that peripheral ghrelin reaches reward nodes and activates the cholinergic-dopaminergic reward link via local GHS-R1A. In support of this, ghrelin passes the blood brain barrier [59], peripherally administered ghrelin increased accumbal dopamine release in rodents [26], [29] and local administration of GHS-R1A antagonists in the VTA blocked peripherally administered ghrelin to increase food intake and to induce reward in rodents [23], [60]. Thus, centrally produced ghrelin [61] may also be of importance for reward regulation. Supportively, we here showed that local LDTg administration of ghrelin also give this dual neurotransmitter release and previous studies show that GHS-R1A is expressed on cholinergic neurons in the LDTg [19].

The present study also show that the ability of ghrelin, administered into the LDTg, to increase accumbal dopamine, but not VTA-acetylcholine, is blocked by perfusion of the unselective nicotinic acetylcholine receptor antagonist, mecamylamine, into the VTA. Previously, alpha3beta2, beta3 and alpha6 nicotinic acetylcholine receptors subtypes in the VTA appear to be critical for this activation [24]. Neurochemical analogies between ghrelin and alcohol are implied since alcohol consumption in rats increases ventral tegmental acetylcholine and accumbal dopamine concomitantly and since alpha3beta2, beta3 and alpha6 nicotinic acetylcholine receptor subtypes in the VTA mediate the reinforcing properties of alcohol [7], [62], [63]. Taken together, this suggests that ghrelin activates GHS-R1A expressed on cholinergic neurons in the LDTg causing a release of ventral tegmental acetylcholine, which via activation of nicotinic receptors in the VTA, increases accumbal dopamine in rats. It should however be emphasized that in addition to the LDTg, GHS-R1A are expressed in both the VTA and N.Acc. [19], [20], [21], raising the possibility that both these areas are involved in the mechanisms by which ghrelin activates the cholinergic-dopaminergic reward link. In the VTA, several mechanisms appear to mediate this effect as nicotinic acetylcholine as well as glutamate receptor antagonists, administered in the VTA, block the ability of ghrelin to active the reward systems, as measured by increased accumbal dopamine, locomotor stimulation and conditioned place preference, and to increase food intake [23], [24], [60]. This raises the possibility that ghrelin administered into the VTA, via GHS-R1A, activates the cholinergic-dopaminergic reward link via nicotinic and glutamatergic mechanisms, however this needs to be further elucidated.

In summary, the present study provides proof of our concept that ghrelin activates the cholinergic-dopaminergic reward link, which is intimately involved in natural- as well as drug-induced reward and also associated with addictive behaviours. These data may have clinical implications since hyperghrelinemia is associated with addictive behaviours including binge eating and alcohol use disorder. Thus, anorectic and bulimic patients of the binge eating type have higher levels of ghrelin in the plasma than their non-binge eating counterparts, and the frequencies of binge eating correlate positively with plasma ghrelin levels [64]. Additionally, the plasma levels of ghrelin are elevated in alcohol-dependent patients [65], [66], albeit this has not been reported in all studies [67]. Hyperghrelinemia has been found in abstinent patients [65], [66], [68], and this is also the case for active ghrelin [69]. Intriguingly, high alcohol craving and likelihood of drink is related to high plasma levels of ghrelin [68], [70]. Given that reward is a part of the addiction processes and that ghrelin signalling has a general role in reinforcement it may be proposed that ghrelin-responsive circuits may serve as a novel pharmacological target for treatment of such addictive behaviours.
